# Brain Tsunamis in Human High-Grade Glioma: Preliminary Observations

**DOI:** 10.3390/brainsci12060710

**Published:** 2022-05-30

**Authors:** Kayli Colpitts, Masoom J. Desai, Michael Kogan, C. William Shuttleworth, Andrew P. Carlson

**Affiliations:** 1 Department of Neurosurgery, University of New Mexico School of Medicine, Albuquerque, NM 87131, USA; kcolpitts@salud.unm.edu (K.C.); mikogan@salud.unm.edu (M.K.); 2 Department of Neurology, University of New Mexico School of Medicine, Albuquerque, NM 87131, USA; mdesai@salud.unm.edu; 3 Department of Neuroscience, University of New Mexico School of Medicine, Albuquerque, NM 87131, USA; bshuttleworth@salud.unm.edu

**Keywords:** spreading depolarization, glioblastoma, glutamate excitotoxicity, high grade glioma

## Abstract

Gliomas make up nearly 40% of all central nervous system tumors, with over 50% of those being high-grade gliomas. Emerging data suggests that electrophysiologic events in the peri-tumoral region may play a role in the behavior and progression of high-grade gliomas. While seizures in the peri-tumoral zone are well described, much larger and slowly propagating waves of spreading depolarization (SD) may potentially have roles in both non-epileptic transient neurologic deficits and tumor progression. SD has only recently been observed in pre-clinical glioma models and it is not known whether these events occur clinically. We present a case of SD occurring in a human high-grade glioma using gold-standard subdural DC ECoG recordings. This finding could have meaningful implications for both clinical symptomatology and potentially for disease progression in these patients. Our observations and hypotheses are based on analogy with a large body of evidence in stroke and acute neurological injury that have recently established SD as cause of transient neurological deficits as well as a fundamental mechanism of ischemic expansion. Whether SD could represent a mechanistic target in this process to limit such progression is a high priority for further clinical investigations.

Mechanisms of infiltration of high-grade glioma are topics of high interest given that their behavior is atypical for most neoplasms [1]. Glioma expansion is characterized by cytotoxic cell death, poorly defined margins, and infiltrating edema formation [2,3]. Discovery of glioma cells in biopsy specimens remote from the enhancing core have challenged the notion that even aggressive surgical resection can play anything more than a palliative role in tumor treatment [3,4,5] and support the concept that physiological events in the surrounding environment rather than simple unregulated mass growth are involved in tumor infiltration. These underlying mechanisms may be one reason for the disappointing lack of significant improvement in patient survival [6] since publication of the Stupp regimen of resection, fractionated radiotherapy and temozolomide [7].

Recent work has focused increasingly on the role that physiological phenomena may play at the infiltrative border of high-grade glioma. Huberfeld and Vecht have described a relationship between seizures and glioma that may be beyond simple association [8]. They note similar epigenetic pathways in seizure development and tumor growth, suggesting that seizures may in fact play a causative role in tumor progression [8]. Seizures and other types of neurological dysfunction can characterize the initial presentation, but this may not be due solely to the mass effect from the tumor, as seizures also predict recurrence of disease [9].

In addition to the role of seizures, a recent preclinical study has implicated massive slow-moving waves of spreading depolarization (SD) in the early stages of glioma development in an immunocompetent murine model [10]. These SD events surprisingly and consistently preceded seizure development. SD is a well described (though under-recognized) neurophysiological behavior that is unlike any normal signaling event. It is characterized by slowly-propagating near complete cellular depolarization which results in cell swelling, massive ionic imbalance, and transient loss of normal signaling capabilities, which is observed as the “spreading depression” described by Leao [11,12,13]. These events require intact neurovascular coupling and metabolic reserve in order to recover, and such recovery is characterized by a massive local increase in blood flow to match the metabolic demand of restoring ionic gradients [14]. These events have been likened to tsunamis since a remote initiation site can spread like a massive wave outward and cause damage even to remote areas, similar to the destruction of a tsunami to low-lying coastal regions.

Since the occurrence of SD has implications for both clinical symptomatology and potentially for disease progression, we sought to explicitly evaluate whether SD could occur in human glioma patients similar to the recent observations in the murine model. We enrolled three human subjects undergoing resection of high-grade glioma in an observational IRB approved study (UNM HRPO# 19-392). Under this IRB approved protocol, we prospectively consented patients to have a subdural electrode strip placed during their clinically indicated surgical resection. Electrode strips were all placed adjacent to the tumor resection bed and removed at bedside, similar to the removal of a surgical drain. We performed intra-operative and post- operative subdural DC ECoG monitoring for up to 3 days post operatively in these subjects and recorded instances of neurological deficits, seizures, and SD.

All three patients presented with episodes of seizures or transient deficits such as extremity weakness and aphasia (see Table 1). All subjects had some electrographic intermittent sharply contoured slowing with periods of frequent spikes and sharps through the monitoring duration, however none had gross clinical deterioration or clinical seizure during the monitoring period. Although all patients presented with seizure or transient neurological deficits, none of these were observed during the monitoring period of our patients.

In one subject, we were able to clearly detect four definite SD with characteristic DC shift and transient suppression of high frequency activity (see Figure 1). These events appeared to originate from the anterior electrodes, closest to the tumor cavity. A total of 59 h, 52 min of recording time was available in this subject, which was notably more than the other two subjects in whom SD was not detected (23 h, 4 m and 26 h, 6 m). In the subject with SD, there were several electrographic seizures throughout monitoring, including several cyclic seizures that occurred prior to the onset of the most significant SD.

While these findings are preliminary, this is an important initial observation to confirm the occurrence of SD in a human subject with high grade glioma as hypothesized by animal data. Although the number of participants is limited, these preliminary observations and the current literature suggests SD could be considered in the progression of high-grade glioma. Further monitoring of patients with longer monitoring periods is of great importance to determine the exact role of SD in high-grade gliomas. If SD occurs outside of the acute perioperative period, it would have additional implications for these patients. First, SD has been shown to be related to transient neurological deficits, most notably demonstrated in a recent series of patients we studied with chronic subdural hematoma, where in one subject aphasia was time-locked to a cluster of recurrent left temporal SD [15]. We also recently reported a similar patient with SD associated with both motor and consciousness deterioration with chronic subdural hematoma, who was treated with memantine and ketamine, stopping the recurrent SD. The neurologic exam returned to normal after cessation of the SD [16]. In addition, clinical deterioration associated with delayed cerebral ischemia has been closely linked to clusters of SD [17]. Many patients with high-grade gliomas present not only with seizures, but also with other non-seizure transient deficits such as numbness and weakness in extremities and aphasia [9]. SD could represent a plausible mechanism for these transient deficits.

Even more intriguing is the possibility that SD could potentially be involved in excitotoxicity and edema progression. Glutamate appears to play a central role in the process of both epileptogenesis and tumor infiltration through multiple mechanisms [18,19]. While the role of specific transporters has been proposed, the physiologic process for this glutamate release is not well established [19,20]. Recent work in other models and disorders demonstrates that SDs could provide a primary source of excitotoxic glutamate. Thus, SDs have emerged as a source of excitotoxic glutamate accumulation in brain slice [21] and in a rat stroke model studies [22], implying that glutamate excitotoxicity is limited to brief periods defined by relatively infrequent, propagating SD waves. This is a major shift from prior assumptions that continuous glutamate “leak” from a damaged infarct core is a source of damaging glutamate. Analogous to the situation with growing ischemic infarcts, it is plausible that glutamate could be released in discrete but massive SD events in glioma patients. The SDs and the immediately following glutamate increase may contribute to making the surrounding native brain tissue more vulnerable to the infiltrative mechanisms of high-grade gliomas.

The second parallel between acute neurological injuries and high-grade glioma centers around vasogenic edema formation. When SD occurs on a background of metabolic compromise (such as ischemic penumbra), it is accompanied by a wave of spreading ischemia [11]. In an elegant study in mice, Mestre demonstrated that SD/spreading ischemia may be the central physiologic mechanism for vasogenic edema formation [23]. While the vasogenic edema is self-limited after stroke, high grade glioma is characterized by extensive, progressive vasogenic edema formation [24]. The cause in this edema formation in the tumor population has not been well established [24], however repeated waves of SD in the peritumoral vulnerable brain could be a plausible explanation.

The parallels of glutamate excitotoxicity and extensive vasogenic edema formation between acute stroke and glioma expansion are therefore worth further investigation. There is evidence that glioma cells may be relatively resistant to these excitotoxic effects [19] and that depolarization events at the margin of the tumor may lead to cell death and perineuronal net loss of normal cells that is observed commonly during tumor progression [10]. One recently proposed mechanism that could play a role in this process by stabilizing glioma cells is through neuron-glioma synapses. Glioma cells in this model were found to have long lasting depolarization that spread extrasynaptically through gap junctions within the tumor cells [25,26,27]. Preventing depolarization increased the survival time of the animals, and optogenetically induced depolarization increased the tumor growth [26,27]. Though the role of SD per-se was not studied in this model, it seems very plausible that the long-lasting depolarization events could be related to the SD observed by Hatcher [10]. If SD in fact plays a potentially pathogenic role in excitotoxicity, infiltration, and peritumoral edema formation, it may add to our understanding of high-grade glioma progression.

In summary, we present the first human case of SD occurring in a patient with high-grade glioma. One potential implication of this finding is as a potential cause of non-seizure transient neurological deficits in such patients. Even more intriguing is the plausible hypothesis, building on recent paradigm shifting findings in ischemic stroke, that SD could play a role in the pathogenesis of tumor progression. Early ischemic expansion after stroke (over days) and malignant tumor expansion of high-grade glioma (over months) may share some pathological processes characterized by glutamate excitotoxicity and vasogenic edema formation. While the initiating factors may differ, it is plausible that SD could represent a common physiologic mechanism of progression. The role and involvement of SD should therefore be studied further in patients with high-grade glioma as this could represent a novel therapeutic target.

## Figures and Tables

**Figure 1:**
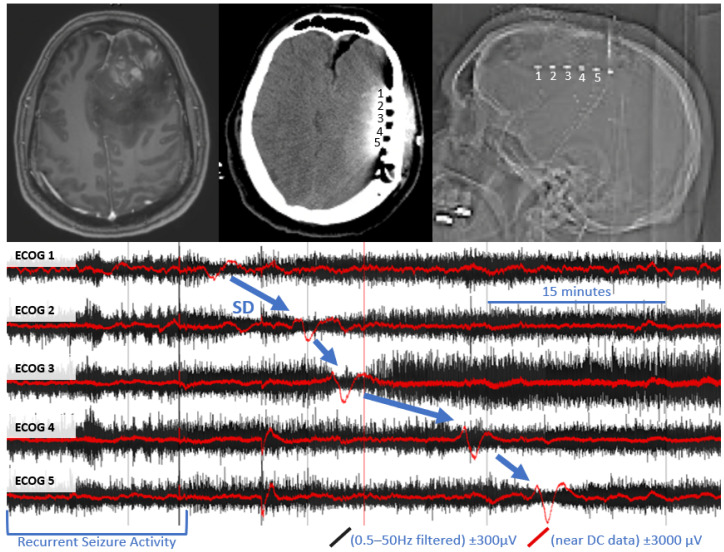
Example of spreading depolarization in a patient with high grade glioma. Upper left panel: pre-operative MRI with and without contrast, demonstrating contrast enhancing left frontal mass with surrounding vasogenic edema. Upper middle panel: post-operative CT, demonstrating the location of the subdural electrode (labeled 1–5) posterior to the tumor resection bed. Upper right panel: scout film from CT also demonstrating the location of the electrode. Bottom panel: referential traces from contacts 1–5 (1 is closest to tumor bed). The black traces represent time compressed, high frequency ECoG data, filtered a 0.5–50 Hz. Overlying red traces represent the same data near DC traces (>0.005 Hz for baseline leveling). A propagating wave of DC shift/slow potential change (in red) that is accompanied by transient suppression of high frequency signaling (in black) is demonstrated (blue arrows).

**Table 1:** Summary of clinical presentation. Onset symptoms were recorded prior to surgical intervention. Doses of pharmacological treatments increased from initial presentation to time of surgical intervention for two of the patients.

	Onset Symptoms	Pathology Diagnosis	Location of Lesion	Maximum Diameter	Extent of Surgical Resection	SD(s) Observed	Pharmacological Treatments
**Patient 1**	Severe headache, extremity weakness, seizures, incontinence	Glioblastoma WHO Grade IV MGMT Promotor Hypermethylated	Right Parietal Lobe	4 cm	Maximal Resection	N	Keppra Dexamethasone
**Patient 2**	Motor aphasia, presyncope, extremity weakness, confusion	Glioblastoma WHO Grade IV	Left Temporal Lobe	4.9 cm	Maximal Resection	N	Keppraa Dexamethasone
**Patient 3**	Headaches, blurred vision, extremity weakness, seizures, incontinence	Glioblastoma WHO Grade IV IDH1 Wild Type MGMT Promotor Hypermethylated	Left Frontal Lobe	11 cm	Subtotal Resection	Y	Keppraa Dexamethasone

## Data Availability

The data presented in this study are available on request from the corresponding author. The data are subject to institutional data sharing oversight.

## References

[1] Watkins S., Sontheimer H. (2012). Unique biology of gliomas: Challenges and opportunities. Trends Neurosci..

[2] Ko C.C., Tai M.H., Li C.F., Chen T.Y., Chen J.H., Shu G., Ko Y.T., Lee Y.C. (2016). Differentiation between Glioblastoma Multiforme and Primary Cerebral Lymphoma: Additional Benefits of Quantitative Diffusion-Weighted MR Imaging. PLoS ONE.

[3] Qual D.F., Joyce J.J. (2017). The microenvironmental landscape of brain tumors. Cancer Cell.

[4] Cheng L., Wu Q., Guryanova O.A., Huang Z., Huang Q., Rich J.N., Bao S. (2011). Elevated invasive potential of glioblastoma stem cells. Biochem. Biophys. Res. Commun..

[5] Kirkpatrick J.P., Sampson J.H. (2014). Recurrent Malignant Gliomas. Semin. Radiat. Oncol..

[6] Poon M.T.C., Sudlow C.L.M., Figueroa J.D., Brennan P.M. (2020). Longer-term (≥2 years) survival in patients with glioblastoma in population-based studies pre- and post-2005: A systematic review and meta-analysis. Sci. Rep..

[7] Stupp R., Mason W.P., van den Bent M.J., Weller M., Fisher B., Taphoorn M.J.B., Belanger K., Brandes A.A., Marsoi C., Bogdahn U. (2005). Radiotherapy plus Concomitant and Adjuvant Temozolomide for Glioblastoma. N. Engl. J. Med..

[8] Huberfeld G., Vecht C.J. (2016). Seizures and gliomas—towards a single therapeutic approach. Nat. Rev. Neurol..

[9] IJerzman-Korevaar M., Snijders T.J., de Graeff A., Teunissen S.C.C.M. (2018). Prevalence of symptoms in glioma patients throughout the disease trajectory: A systematic review. J. Neurooncol..

[10] Hatcher A., Yu K., Meyer J., Aiba I., Deneen B., Noebels J.L. (2020). Pathogenesis of peritumoral hyperexcitability in an immunocompetent CRISPR-based glioblastoma model. J. Clin. Investig..

[11] Dreier J.P. (2011). The role of spreading depression, spreading depolarization and spreading ischemia in neurological disease. Nat. Med..

[12] Dreier J.P., Fabricius M., Ayata C., Sakowitz O.W., Shuttleworth C.W., Dohmen C., Graf R., Vajkoczy P., Helbok R., Suzuki M. (2017). Recording, analysis, and interpretation of spreading depolarizations in neurointensive care: Review and recommendations of the COSBID research group. J. Cereb. Blood Flow Metab..

[13] Leao A.A. (1944). Spreading depression of activity in the cerebral cortex. J. Neurophysiol..

[14] Ayata C., Lauritzen M. (2015). Spreading Depression, Spreading Depolarizations, and the Cerebral Vasculature. Physiol. Rev..

[15] Mohammad L.M., Abbas M., Shuttleworth C.W., Ahmadian R., Bhat A., Hill D.A., Carlson A.P. (2020). Spreading depolarization may represent a novel mechanism for delayed fluctuating neurological deficit after chronic subdural hematoma evacuation. J. Neurosurg..

[16] Reinhart K.M., Humphrey A., Brennen K.C., Carlson A.P., Shuttleworth C.W. (2021). Memantine Improves Recovery After Spreading Depolarization in Brain Slices and can be Considered for Future Clinical Trials. Neurocrit. Care.

[17] Dreier J.P., Woitzik J., Fabricius M., Bhatia R., Major D., Drenckhahn C., Lehmann T.-N., Sarrafzadeh A., Willumsen L., Hartings J.A. (2006). Delayed ischaemic neurological deficits after subarachnoid haemorrhage are associated with clusters of spreading depolarizations. Brain.

[18] Buckingham S.C., Campbell S.L., Haas B.R., Montana V., Robel S., Ogunrinu T., Sontheimer H. (2011). Glutamate release by primary brain tumors induces epileptic activity. Nat. Med..

[19] De Groot J., Sontheimer H. (2011). Glutamate and the biology of gliomas. Glia.

[20] Campbell S.L., Buckingham S.C., Sontheimer H. (2012). Human glioma cells induce hyperexcitability in cortical networks. Epilepsia.

[21] Aiba I., Shuttleworth C.W. (2012). Sustained NMDA receptor activation by spreading depolarizations can initiate excitotoxic injury in metabolically compromised neurons. J. Physiol..

[22] Hinzman J.M., DiNapoli V.A., Mahoney E.J., Gerhardt G.A., Hartings J.A. (2015). Spreading depolarizations mediate excitotoxicity in the development of acute cortical lesions. Exp. Neurol..

[23] Mestre H., Du T., Sweeney A.M., Liu G., Samson A.J., Peng W., Mortensen K.N., Stæger F.F., Bork P.A.R., Bashford L. (2020). Cerebrospinal fluid influx drives acute ischemic tissue swelling. Science.

[24] Lin Z.-X. (2013). Glioma-related edema: New insight into molecular mechanisms and their clinical implications. Chin. J. Cancer.

[25] Barria A. (2019). Dangerous liaisons as tumours form synapses. Nature.

[26] Venkataramani V., Tanev D.I., Strahle C., Studier-Fischer A., Fankhauser L., Kessler T., Körber C., Kardorff M., Ratliff M., Xie R. (2019). Glutamatergic synaptic input to glioma cells drives brain tumour progression. Nature.

[27] Venkatesh H.S., Morishita W., Geraghty A.C., Silverbush D., Gillespie S.M., Arzt M., Tam L.T., Espenel C., Ponnuswami A., Ni L. (2019). Electrical and synaptic integration of glioma into neural circuits. Nature.

